# Estimation of prevalence of autoimmune diseases in the United States using electronic health record data

**DOI:** 10.1172/JCI178722

**Published:** 2024-12-12

**Authors:** Aaron H. Abend, Ingrid He, Neil Bahroos, Stratos Christianakis, Ashley B. Crew, Leanna M. Wise, Gloria P. Lipori, Xing He, Shawn N. Murphy, Christopher D. Herrick, Jagannadha Avasarala, Mark G. Weiner, Jacob S. Zelko, Erica Matute-Arcos, Mark Abajian, Philip R.O. Payne, Albert M. Lai, Heath A. Davis, Asher A. Hoberg, Chris E. Ortman, Amit D. Gode, Bradley W. Taylor, Kristen I. Osinski, Damian N. Di Florio, Noel R. Rose, Frederick W. Miller, George C. Tsokos, DeLisa Fairweather

**Affiliations:** 1 Autoimmune Registry Inc., Guilford, Connecticut, USA.; 2 Division of Bioinformatics, Department of Population and Public Health Sciences, Keck School of Medicine and; 3 Department of Dermatology, Keck School of Medicine, University of Southern California, Los Angeles, California, USA.; 4 University of Florida Health, Gainesville, Florida, USA.; 5 Department of Neurology, Massachusetts General Hospital, Boston, Massachusetts, USA.; 6 Research Information Systems and Computing, Mass General Brigham, Somerville, Massachusetts, USA.; 7 Department of Neurology, University of Kentucky Medical Center, Lexington, Kentucky, USA.; 8 Department of Population Health Sciences, Weill Cornell Medicine, New York, New York, USA.; 9 Georgia Tech Research Institute, Atlanta, Georgia, USA.; 10 Institute for Informatics, Data Science, and Biostatistics, Washington University School of Medicine, St. Louis, Missouri, USA.; 11 Institute for Clinical and Translational Science, University of Iowa, Iowa City, Iowa, USA.; 12 Clinical & Translational Science Institute, Medical College of Wisconsin, Milwaukee, Wisconsin, USA.; 13 Department of Cardiovascular Medicine, Mayo Clinic, Jacksonville, Florida, USA.; 14 Center for Clinical and Translational Science and; 15 Mayo Clinic Graduate School of Biomedical Sciences, Mayo Clinic, Rochester, Minnesota, USA.; 16 Harvard Medical School, Boston, Massachusetts, USA.; 17 National Institute of Environmental Health Sciences, National Institutes of Health, Research Triangle Park, North Carolina, USA.; 18 Department of Medicine, Beth Israel Deaconess Medical Center, Boston, Massachusetts, USA.; 19 Department of Immunology, Mayo Clinic, Jacksonville, Florida, USA.

**Keywords:** Autoimmunity, Autoimmune diseases, Epidemiology, Sex hormones

## Abstract

**BACKGROUND:**

Previous epidemiologic studies of autoimmune diseases in the US have included a limited number of diseases or used metaanalyses that rely on different data collection methods and analyses for each disease.

**METHODS:**

To estimate the prevalence of autoimmune diseases in the US, we used electronic health record data from 6 large medical systems in the US. We developed a software program using common methodology to compute the estimated prevalence of autoimmune diseases alone and in aggregate that can be readily used by other investigators to replicate or modify the analysis over time.

**RESULTS:**

Our findings indicate that over 15 million people, or 4.6% of the US population, have been diagnosed with at least 1 autoimmune disease from January 1, 2011, to June 1, 2022, and 34% of those are diagnosed with more than 1 autoimmune disease. As expected, females (63% of those with autoimmune disease) were almost twice as likely as males to be diagnosed with an autoimmune disease. We identified the top 20 autoimmune diseases based on prevalence and according to sex and age.

**CONCLUSION:**

Here, we provide, for what we believe to be the first time, a large-scale prevalence estimate of autoimmune disease in the US by sex and age.

**FUNDING:**

Autoimmune Registry Inc., the National Heart Lung and Blood Institute, the National Center for Advancing Translational Sciences, the Intramural Research Program of the National Institute of Environmental Health Sciences.

## Abstract 2

This paper estimates the prevalence of all autoimmune disease in the US, sex- and age-adjusted, with individual estimates for 105 autoimmune diseases.

## Graphical abstract

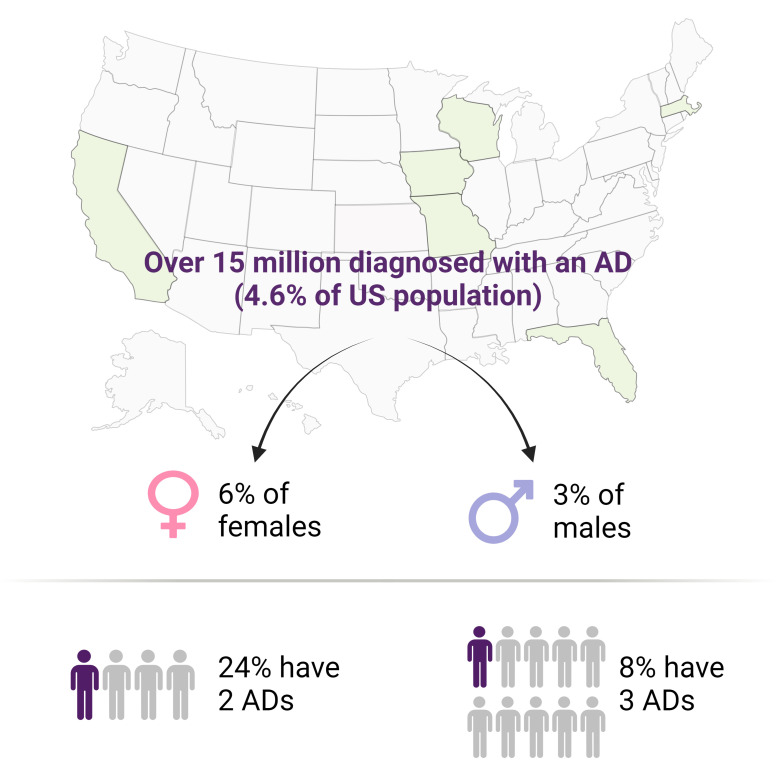

## Introduction

Autoimmune diseases are a diverse group of chronic inflammatory pathologies marked by a dysfunctional innate and adaptive immune system after exposure to proinflammatory environmental agents resulting in subsequent end-organ damage that lead to clinical disease manifestations (1). Although studies of the prevalence and incidence of individual autoimmune diseases have been reported, the prevalence of autoimmune diseases as a class has only been estimated 5 times to date, most recently in 2023 in the United Kingdom (Table 1) (2–6). Many challenges exist to obtain accurate data on the prevalence of all autoimmune diseases, including the lack of an international consensus on the definition of autoimmune disease and which specific entities fall into this category (7).

Precedence for classifying diseases into major categories can been seen in cancer (8), cardiovascular diseases (9), and organ-specific diseases including the skin (10), respiratory (11), and digestive systems (12). Prevalence statistics for individual diseases provide context to interpret test results used to diagnose patients (13). Prevalence statistics by disease class can help assess the burden of these diseases on a population. There is a need to assess the prevalence of autoimmune diseases as a class to fully appreciate their impact on society, where many rare autoimmune conditions may otherwise be ignored.

Knowledge of disease class prevalence (i.e., autoimmune diseases) is also important to raise public awareness of autoimmune diseases in general, which helps channel funding to individual autoimmune diseases and assists in the recognition of rare autoimmune diseases. As highlighted in a recent National Academy of Sciences, Engineering, and Medicine report, research and public awareness efforts for autoimmune diseases have focused almost exclusively on a limited number of autoimmune diseases including inflammatory bowel disease, multiple sclerosis, type I diabetes, rheumatoid arthritis (RA), and systemic lupus erythematosus (14). The American Heart Association (AHA) describes deaths and other outcomes for all cardiovascular diseases before breaking data down by categories of heart disease (15), and these data are used by the AHA for public awareness campaigns to emphasize the importance of clinical and research efforts to decrease heart disease. Similarly, prevalence and incidence data on cancer as a class provided by the National Cancer Institute’s Surveillance, Epidemiology and End Results Program (SEER, https://seer.cancer.gov) and the American Cancer Society (https://www.cancer.gov) are used to emphasize the need to research cures for cancer. Thus, knowing the overall prevalence of diseases by class is an important component of research and public health awareness efforts in the US. The research community and the public should have access to similar data for autoimmune diseases in the US as they do for heart disease and cancer. This study is the first to our knowledge to examine a large number of autoimmune diseases in the US using nationwide data.

Another reason to gather information on autoimmune diseases as a group is that, due to shared environmental or genetic risk factors, individuals quite frequently suffer from multiple autoimmune conditions (16, 17). For example, polymorphisms in certain immune genes have been found to occur in several autoimmune diseases (18), which provides a possible explanation for the occurrence of multiple autoimmune diseases in the same individual. Research strategies that count individual autoimmune diseases and then aggregate those statistics for multiple autoimmune diseases count individuals more than once and thereby might overstate prevalence. This is a common issue found in metadata assessments of prevalence that makes estimations of prevalence over time difficult.

Finally, there is recent evidence suggesting that the prevalence of biomarkers, such as antinuclear antibodies (19), has increased for at least some autoimmune diseases, and the scientific community and the public need to know whether this increase is associated with a parallel increase in the incidence and prevalence of autoimmune diseases (20, 21). There is an urgency to develop approaches to compute the prevalence of autoimmune diseases that can be replicated longitudinally. Therefore, we aimed to provide an update on the prior estimates by providing a current overall autoimmune disease prevalence estimate in the US according to sex and age as well as for 105 autoimmune diseases using electronic health record (EHR) data.

## Results

In order to determine the prevalence of autoimmune diseases in the US we selected 105 diseases that were listed in the textbook “The Autoimmune Diseases” by Rose and MacKay, 5th Edition (1), that had substantiative evidence of an autoimmune pathology (Supplemental Table 1; supplemental material available online with this article; https://doi.org/10.1172/JCI178722DS1). Our list included all autoimmune diseases for which there is evidence in the literature that self-reactive T cells and/or antibodies contribute to or cause the disease (22). We included cis-females (referred to hereafter as females) and cis-males (referred to hereafter as males) with no age restriction, according to gender information provided in the EHR. A list of diseases that lack clear evidence for an autoimmune pathology, but are often categorized or described as autoimmune, is included in Supplemental Table 2, along with published and computed prevalence for thoroughness; however, these conditions were not included in our prevalence estimates.

**Figure 1:**
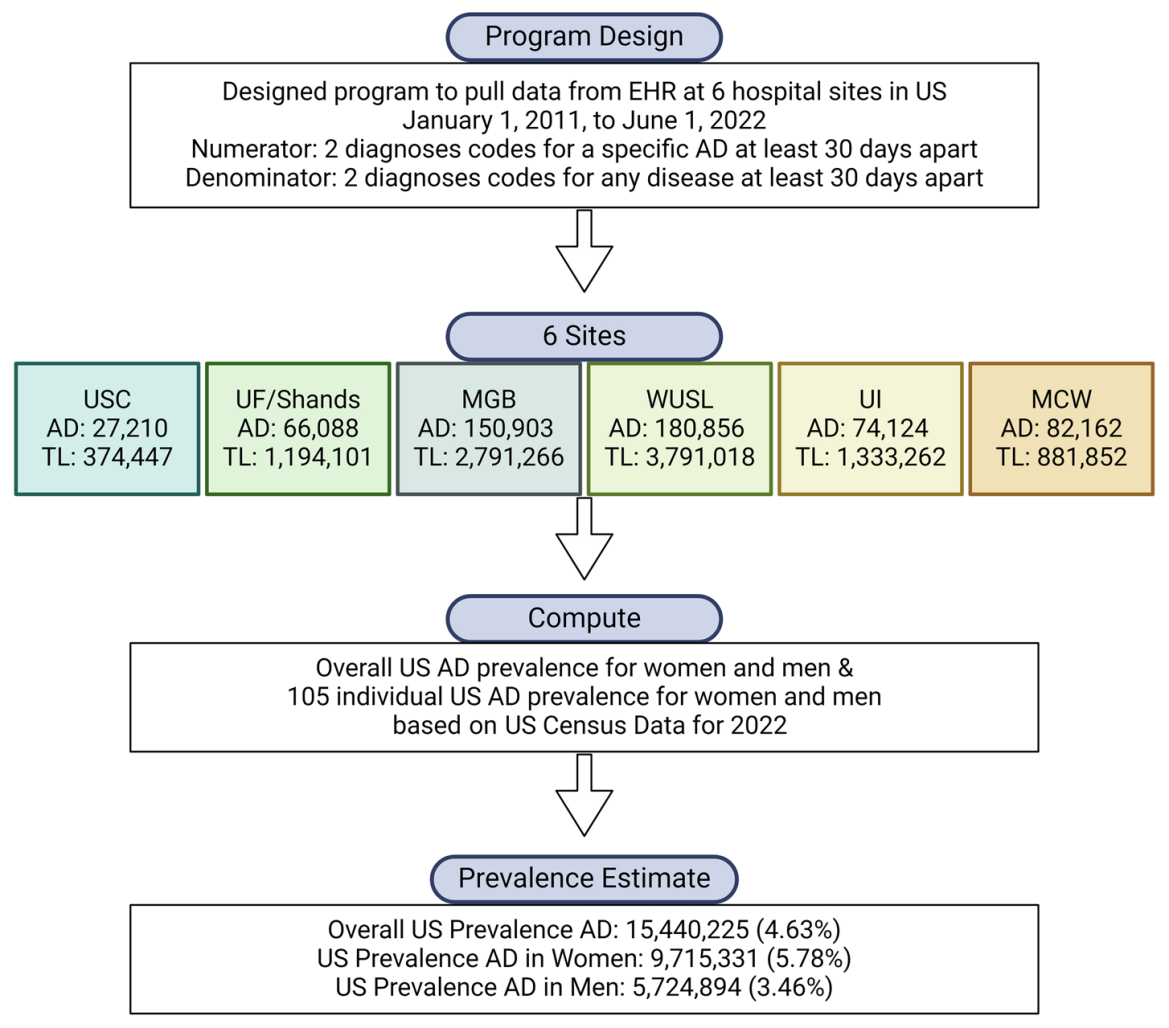
A flow chart of the study design. A total (TL) of 10,365,946 individuals were identified from the electronic health record (EHR) from January 1, 2011, to June 1, 2022, from 6 healthcare sites in the US based on a program that identified patients with 2 diagnoses codes for any disease at least 30 days apart (denominator). From this total, 581,343 individuals were identified with 1 of 105 specific autoimmune diseases (ADs) based on 2 diagnoses codes at least 30 days apart (numerator) in the EHR. Overall AD prevalence for women and men was computed based on US Census Data for 2022. The 6 healthcare sites included University of Southern California (USC), University of Florida (UF)/ Shands, Mass General Brigham (MGB), Washington University of St. Louis (WUSL), University of Iowa (UI), and the Medical College of Wisconsin (MCW). The image was designed using BioRender.

Between January 1, 2011 and June 1, 2022, we identified a total of 581,343 individuals from 6 medical systems across the US serving a population of 10,365,946 that were diagnosed with at least 1 of the 105 autoimmune diseases considered in this study (Figure 1 and Table 2). Extending these statistics to an estimated US population of 333.3 million in 2022 (23) (Supplemental Table 3) gives an overall computed prevalence of 15,440,225 individuals (95% CI 15,437,949–15,442,501), or 4.6% of the US population with an autoimmune disease. The prevalences of each of the 105 individual autoimmune diseases by sex are shown in Supplemental Table 1, along with the published estimated prevalence as of January 1, 2022. For 22 of the 105 diseases, there were no patients who met the requirements for inclusion, and there were 9 diseases for which the patient counts were below 10 and therefore estimates have not been reported. The overall estimated prevalence for females was 9,715,331 (95% CI 9,680,412–9,750,250) or 5.8% of the US female population, and for males was 5,724,894 (95% CI 5,695,208–5,754,578) or 3.5% of the US male population (Table 2).

**Figure 2:**
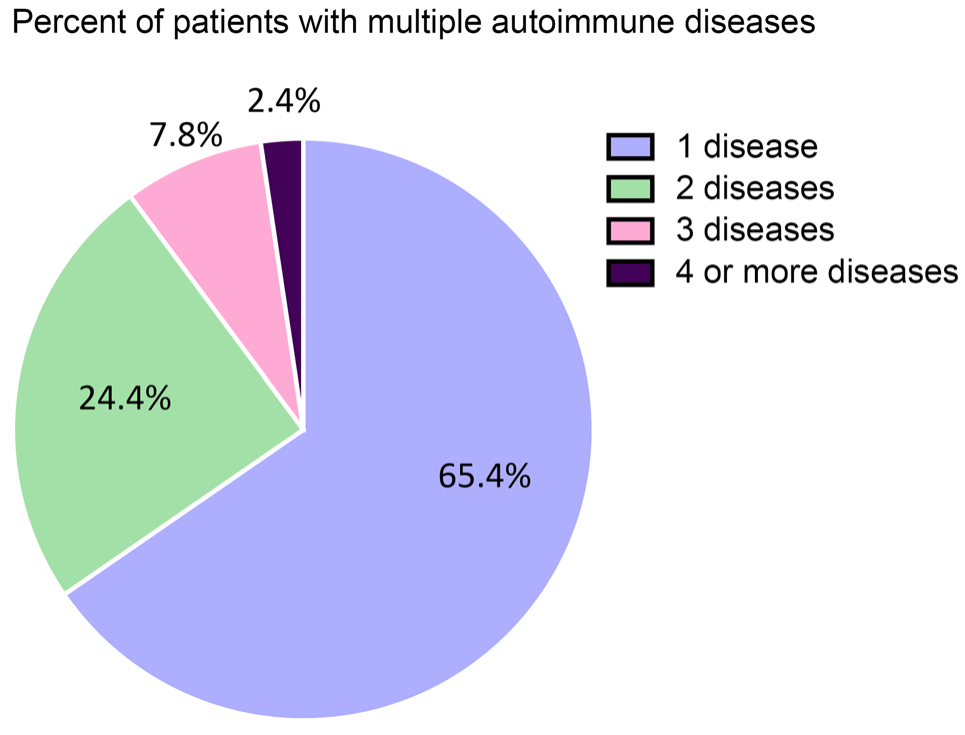
Prevalence of multiple autoimmune diseases. Individuals with 1 autoimmune disease are known to often suffer from another autoimmune condition. Research strategies that count individual autoimmune diseases and then aggregate those statistics for multiple autoimmune diseases count individuals more than once and thereby might overstate prevalence. This figure reports the frequency of multiple autoimmune diseases in this study, indicating that this could be an issue in certain prevalence estimates and indicates how often they cooccur.

As expected, most patients diagnosed with autoimmune diseases were female (63%) compared to male (37%) for an overall sex ratio of 1.7:1 female-to-male. The number of individuals not reporting a sex of male or female was under 20 for many diseases at many of the sites in this study and so is not reported to protect patient privacy. Additionally, 65% of patients had 1 autoimmune disease whereas 24% had 2, 8% had 3, and 2% had 4 or more autoimmune diseases (Figure 2 and Supplemental Table 4). The top 20 autoimmune diseases based on prevalence are listed in Table 3 with RA, psoriasis, type I diabetes mellitus, Graves’ disease, and autoimmune thyroiditis being the top 5. Interestingly, 17 of the top 20 autoimmune diseases occurred more often in females than males. The top autoimmune diseases in females or males based on sex ratio are listed in Tables 4 and 5, respectively.

## Discussion

In this study we developed a new tool to estimate the prevalence of autoimmune disease in the US. Our methodology offers the following advantages: (a) The entire analysis can be run at any site that has data in the widely used Observational Medical Outcomes Partnership (OMOP) model; (b) the tool is easy to use and generally takes a few hours to run; (c) the diseases selected for inclusion can be easily modified; and (d) the tool can be modified to add additional parameters such as medications and labs, to improve diagnostic specificity and sensitivity and for individual research purposes.

Our estimate of over 15 million, or 4.6%, individuals with autoimmune disease in the US is below some commonly quoted estimates of 7%–10% (see Table 1). Our selection criteria that required 2 diagnosis codes at least 30 days apart aimed to reduce counting individuals that were being investigated but had not yet been diagnosed with an autoimmune disease. Several estimates of the prevalence of autoimmune disease have been aggregates of meta-analyses of disease-specific prevalence data, which likely over estimates prevalence due to double counting, since a patient with more than 1 disease is counted in more than 1 of the disease estimates.

We confirmed an overall sex ratio for autoimmune diseases of around 1.7:1 female-to-male. We have provided prevalence by sex for 105 individual autoimmune diseases (Supplemental Table 1). These data are needed to better understand the impact of individual autoimmune diseases by biological sex and to support the need for clinical and basic research examining overall autoimmune and disease-specific mechanisms. Research in autoimmune diseases has not kept pace with advances in other disease categories like cancer and heart disease because of relatively lower funding levels and a paucity of specific data for the US population.

Several previous studies found that patients with 1 autoimmune disease are more likely to develop another autoimmune disease (16, 17). However, there has been a lack of data on the prevalence of cooccurrence of autoimmune diseases overall in the US. In this study we show that as many as 24% of patients are diagnosed with 2 autoimmune diseases and 2% have 4 or more autoimmune diseases concurrently. More research is needed to understand which autoimmune diseases cooccur and if common mechanisms can be targeted with improved diagnostic tests and therapies.

There are a number of limitations to our study. The use of EHR data to determine who has an autoimmune disease is complicated by several factors. Since the diagnosis of a given autoimmune disease is rarely, if ever, contingent only on the presence of clear biomarkers, autoimmune disease codes in the EHR might not be accurate (24). Many patients have diagnoses that are subsequently refined or completely changed as their symptoms and clinical findings evolve (25–27). Some diseases can be caused by autoimmune or nonautoimmune processes. An example would be the diagnosis of type 1 diabetes mellitus in a patient who has undergone a total pancreatectomy (28). We could also miss patients with a single diagnosis code since we only count patients with at least 2 diagnosis codes. It is also known that autoimmune diseases evolve over time and involve nonspecific clinical signs and symptoms that can mimic other diseases that may result in an underdiagnosis of many of these diseases. Rare diseases, such as antisynthetase syndrome and IgG4-related disease, lack specific ICD-10 codes (29). Though our analysis uses Systematized Nomenclature of Medicine (SNOMED) codes, which do exist for these diseases, we know that EHR data at the sites we studied, which use ICD-10 coding, will not identify these patients. Using broader disease names, such as “myositis” to capture antisynthetase syndrome, however, captures too many patients who do not have this autoimmune disease. Therefore, we included a category of “Autoimmune Disease Not Otherwise Specified” which will capture some of these diseases. Additionally, our dataset was based on data from academic medical centers. Such systems include more specialists and fewer general practitioners, leading to possible selection bias. Coding error is another limitation: type 2 diabetics are often miscoded for type 1, leading to inflated values for that condition. Another limitation is that patient death is not typically recorded consistently in EHR systems, so patients who died during the study period will be counted in the numerator. Since these patients are also included in the denominator, this limitation should not have a significant impact on overall prevalence statistics. Also in the US, individuals move location frequently and so it is possible that the same patient could be counted at more than 1 location. However, the 6 sites in this study are in diverse locations which should reduce this error. In spite of these limitations, we believe that the use of a common data model and methodology for all conditions provides support for the accuracy of our estimate, and the software used to compute our estimates can be improved over time as these many limitations are addressed. And, finally, a number of the conditions in our list of autoimmune diseases may not be considered by all investigators to have sufficient evidence to name them autoimmune diseases. For a conservative approach, we included diseases discussed in the textbook “The Autoimmune Diseases” edited by Rose and Mackay (1). However, we fully acknowledge that some conditions may be considered ‘autoinflammatory’ or simply inflammatory conditions. Our goal was to provide data on prevalence by sex for individual autoimmune diseases that may help move the field forward in order to better address these and other issues in the field. Our development of a relatively simple tool now made available freely to the clinical and research community will hopefully fulfill this goal.

### Conclusions.

We developed a new analysis tool to determine the overall and individual prevalence of autoimmune diseases in the US or other countries. Using this tool and data from the EHR of 6 major medical systems in the US, we estimated that autoimmune disease affected over 15 million individuals in the US in 2022, which is 4.6% of the population. Females represented 63% of those with autoimmune disease, and males 37%, a sex ratio of 1.7:1 female-to-male. We report high levels of comorbid autoimmune diseases with 24% of patients with autoimmune disease diagnosed with 2 autoimmune diseases and 8% with 3. Accurate data on the prevalence of autoimmune diseases as a category of disease and for individual autoimmune diseases are needed to further clinical and basic research to improve diagnosis, biomarkers, and therapies for these diseases, which substantially impact the US population.

## Methods

### Sex as a biological variable.

Our study examined sex as a biological variable. We included cis-females (referred to as females) and cis-males (referred to as males) with no age restriction, according to gender information provided in the EHR.

### Data sources.

In this observational study, we obtained EHR data from January 1, 2011, to June 1, 2022 from the University of Southern California Health System (USC), a large multispecialty health system with 2 inpatient tertiary care centers and multiple outpatient specialty clinics across the Los Angeles area; the University of Florida and Shands Health System (UF/Shands), an academic medical network with 11 hospitals and numerous outpatient clinics located in Florida; Mass General Brigham Health System (MGB), a Boston-based nonprofit hospital and physician network; University of Iowa Health Care, the only academic health system in the state that is centrally located in Iowa City, which used Iowa Health Data Resource (30); Medical College of Wisconsin (MCW), a private academic medical center with extensive clinical partnerships across Wisconsin; and Washington University School of Medicine in St. Louis (WUSTL), a private research university partnered with Barnes-Jewish Hospital (Figure 1).

### Study population.

For the denominator, we included patients that had at least 2 diagnoses of any disease at least 30 days apart (the denominator algorithm) (Figure 1). For the numerator, a patient was determined to have a diagnosis of an autoimmune disease if they had at least 2 diagnoses codes for the disease at least 30 days apart (the numerator algorithm). We examined EHR records collected between January 1, 2011 and June 1, 2022. We describe considerations for this analysis strategy below and acknowledge that different approaches affect prevalence outcomes. We want to emphasize, however, that a goal of this manuscript was to provide a program that is freely available for clinicians and researchers to use their own strategies and datasets to arrive at overall and individual US autoimmune disease prevalence estimates.

To test the accuracy of our algorithm, we conducted sensitivity analyses to determine how changing the number of diagnosis codes and the number of days between diagnosis codes (the date window) affected both the numerators and denominators used in our prevalence estimate (Supplemental Tables 5–7). Because the EHR is used for billing purposes in the US, a patient may receive a provisional diagnosis of an autoimmune disease to justify ordering tests to rule out the disease (31). While provisional diagnoses are also used in other countries, they are not required for billing purposes, whereas the US medical system makes such diagnoses a financial requirement (32). Thus, the use of a single diagnostic code to classify a patient as diagnosed with a disease will likely be an inaccurate source for determining prevalence. Supplemental Table 5 demonstrates that use of a single diagnosis code (0 date window) would overstate case counts by 31%–53% (average 41%) if 6 diseases were analyzed.

To investigate the effect of changing the denominator we found that using 2 diagnosis codes and a 30-day window gave a prevalence estimate of 5.9%, while other date windows ranging from 60–720 produced prevalence estimates of around 6.1%–6.5% (Supplemental Table 6). Thus, the prevalence gets larger as the denominator gets smaller with larger date windows because the algorithm catches fewer people. The percent change in prevalence by altering the denominator from 0–30 days or more was around 18% (Supplemental Table 7). However, the prevalence calculated using 2 codes over increasing date windows varied only by a small percentage, indicating that a 30-day date window was a valid and conservative estimate of prevalence (Supplemental Table 7). Based on these analyses, we required 2 diagnostic codes over a minimum time period (the date-window) of 30 days to classify patients as being diagnosed with a specific autoimmune disease. A study by Chung et al. (22) in 2013 also found that the use of 2 diagnostic codes provided improved specificity when using EHR data to identify patients diagnosed with RA. A code of “Autoimmune disease not otherwise classified” plus a specific disease code recorded 30 or more days later also qualified a patient as being diagnosed with a specific autoimmune disease.

To further validate the denominator, and the algorithm generally, we implemented an algorithm for RA developed by researchers at Harvard Medical School for use on the Electronic Medical Records and Genomics (eMERGE) network, a national network organized and funded by the National Human Genome Research Institute. The algorithm, posted on the Phenotype KnowledgeBase (PheKB) as Phenotype 585, is a machine-learning logistic regression model that uses a combination of log-weighted factors to classify patients with and without RA (https://phekb.org/phenotype/rheumatoid-arthritis-ra). The area under the receiver operating curve (AUROC) for the algorithm is 0.95 (see previous URL).

When we ran the eMERGE algorithm on the USC dataset, the program classified 2,552 patients with RA. Our denominator algorithm (2 diagnosis codes at least 30 days apart) computed the USC denominator as 375,253 for a prevalence of 6.8% (Supplemental Table 8). Extending to the US population gave an estimated prevalence of 2,264,648, which is within a published estimated prevalence for RA of 1,099,890 to 2,633,070 (33). Using our algorithm and sex- and age-adjusted data from USC estimates a prevalence of 2,586,344 individuals or 7.8%. Our algorithm across all 6 sites for RA estimates a prevalence of 2,580,060 individuals or 7.7% (Supplemental Table 8).

The date of death is not well tracked in electronic medical records. For sites that provided these data, the algorithm removed the patients. However, at sites without the date of death, patients remain in both the numerator and denominator, so death does not materially alter the prevalence estimate.

### Selection of autoimmune diseases.

The list of 105 autoimmune diseases included in this study was based on the textbook, “The Autoimmune Diseases” by Rose and MacKay, 5th Edition (1), with addition of select autoimmune diseases to establish a list of diseases for which substantive published evidence exists (Supplemental Table 1). Our list included all autoimmune diseases for which there is evidence in the literature that self-reactive T cells and/or antibodies contribute to or cause the disease (22). A list of diseases that lack this evidence, but are often categorized as autoimmune, is included in Supplemental Table 2, along with published and computed prevalence, for thoroughness; however, these conditions were not included in our autoimmune disease prevalence estimates.

### Statistics.

The data were transformed into the Observational Medical Outcomes Partnership (OMOP) model by each institution’s local information technology personnel, and ICD-9 and ICD-10 codes were transformed to the Systematized Nomenclature of Medicine (SNOMED) coding system.

Since our goal was to assess the prevalence of all autoimmune diseases in the US using a standardized and replicable methodology, we sought an algorithm for computing the numerators that met the following criteria: (a) the algorithm is applicable across all autoimmune diseases without being more selective for some diseases than others; (b) the algorithm can operate at many health systems, not just those with a specific EHR system; (c) the algorithm can be run repeatedly so that changes in statistics can be tracked longitudinally; and (d) the algorithm can serve as a basis for more complete algorithms in the future (for example, algorithms that include medications and lab tests in the EHR, as well as notes).

Projecting the site-based prevalence estimates from individual sites to the US population required a denominator for the 6 sites’ populations. Computing a denominator using EHR data has challenges. In the US, EHR data are siloed by healthcare organizations, but patients can cross from one organization to another for their care, especially for emergency visits. Healthy patients may not seek care at all, or when they do, they may go to consumer-oriented facilities outside of the healthcare organizations (e.g., CVS Minute Clinics). Finally, females use the health system more than males (34), which may bias the dataset because females are known to be affected by autoimmune disease more often than males.

To project age- and sex-adjusted prevalence, we stratified the numerator and denominator into 4 age groups across 2 sex categories. Numerator and denominators for each site were used to compute an age-sex ratio, and that ratio was applied to the corresponding age-sex population based on US Census Data for 2022. We then combined the prevalence projections for all 8 age and sex categories into a total projected prevalence for all diseases for each site (disease specific age- and sex-adjusted values have also been computed for validations that appear elsewhere in the paper).

The software program, made freely available to the research community and included in Supplementary Materials, allows the date-windows for the numerator and denominator to be changed independently to allow refinement of this analysis by other investigators.

### Study approval.

Retrospective review of the demographic and clinical data from the EHR reported in this manuscript was approved by the Institutional Review Board (IRB) of each site. The need for written informed consent was waived by each IRB. Initial development of the algorithms was performed at the University of Southern California under IRB HS-20-00902. The IRB at Mass General Brigham determined on 7/11/2023 that the use of deidentified data made the project nonhuman-subject research and the need for an IRB was waived (REDCap ID #691). Work at the University of Florida/ Shands Health System was conducted under IRB 202201755. The IRB at University of Iowa determined the project (IRB# 202403419) was not human subject research on 03/21/24. The Medical College of Wisconsin IRB reviewed the study (ID PRO00051359) on 6/11/2024 and determined it did not meet criteria for human subject research. The IRB at Washington University in St. Louis’ Research Data Core Repository determined that the project (IRB #201607071) reported only summary statistics and did not constitute human subject research. The research conformed to the principles outlined in the Declaration of Helsinki.

### Data availability.

The program code used to generate the data for the manuscript is included in Supplemental Material and is made freely available to the research community provided they acknowledge the manuscript source. The code is modifiable for future studies. All data generated in the study are provided in the manuscript and Supplemental Data Values files. All questions regarding the study and program code should be directed to the cosenior authors of the study.

## Author contributions

AHA conceived the project and directed the study with input from all authors. AHA, MGW, and NRR were involved in study design. NB provided study oversight. MA, XH, JSZ, and CDH provided data access. AHA conducted data analysis. AHA and DF wrote the manuscript. AHA, IH, NB, SC, ABC, LMW, GPL, XH, SNM, CDH, JA, MGW, JSZ, EMA, MA, PROP, AML, HAD, AAH, CEO, ADG, BWT, KIO, DND, NRR, FWM, GCT, and DF were involved in data interpretation and editing the manuscript.

## Supplementary Material

Supplemental data

ICMJE disclosure forms

Supplemental data set 1

## Figures and Tables

**Table 4:**
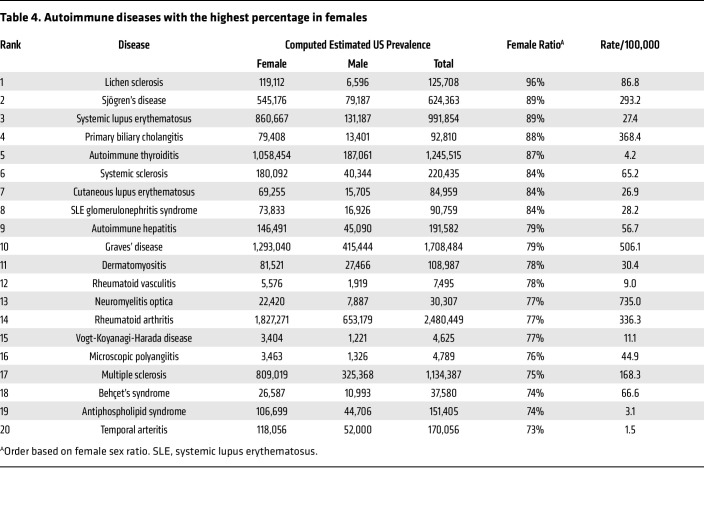
Autoimmune diseases with the highest percentage in females

**Table 3:**
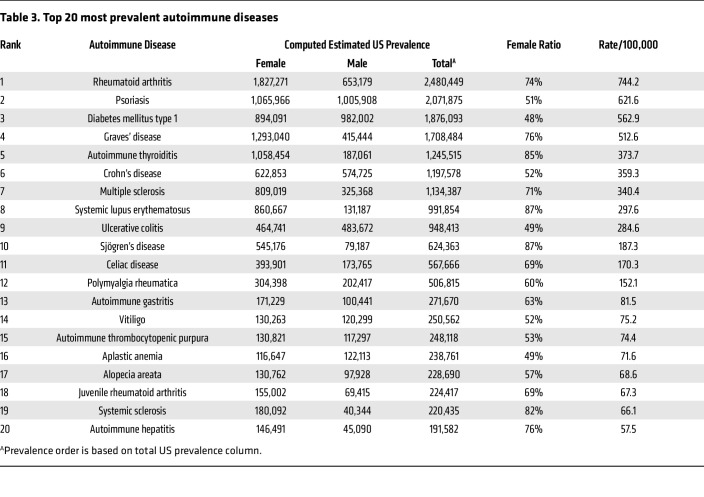
Top 20 most prevalent autoimmune diseases

**Table 2:**
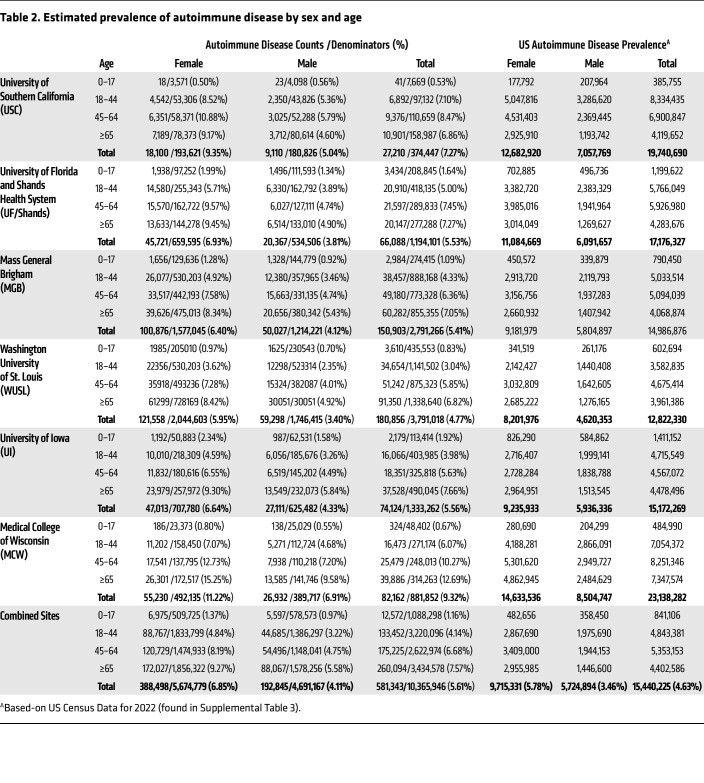
Estimated prevalence of autoimmune disease by sex and age

**Table 1:**
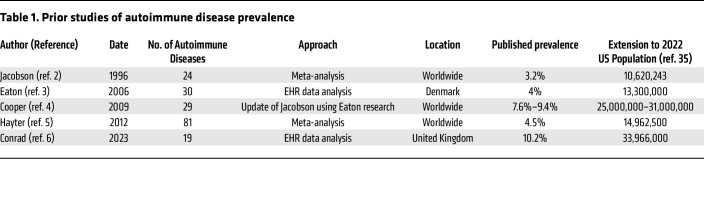
Prior studies of autoimmune disease prevalence

**Table 5:**
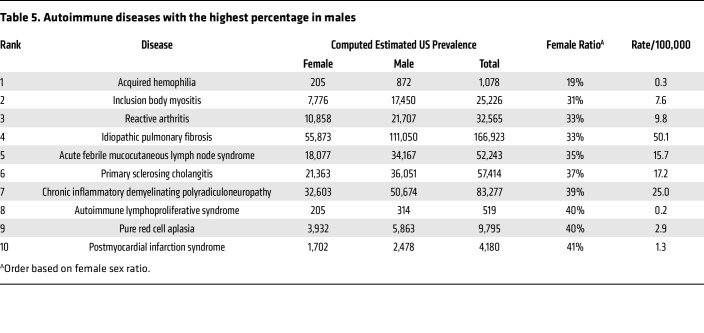
Autoimmune diseases with the highest percentage in males

## Version 1 12/12/2024

In-Press Preview

## Version 2 02/17/2025

Electronic publication
