# Supplementary material for: Estimation of prevalence of autoimmune diseases in the United States using electronic health record data
Source: J Clin Invest. 2024 Dec 12;135(4):e178722. doi: 10.1172/JCI178722 (PMC11827834; doi:10.1172/JCI178722)
Supplement: Supplemental data [file jci-135-178722-s226.pdf]

Supplemental Data

Estimation of prevalence of autoimmune diseases in the United States using electronic health record data

Aaron H. Abend[1$], Ingrid He[1], Neil Bahroos[2], Stratos Christianakis[2], Ashley B. Crew[3], Leanna M. Wise[2], Gloria P. Lipori[4], Xing He[4], Shawn N. Murphy[5], Christopher D. Herrick[6], Jagannadha Avasarala[7], Mark G. Weiner[8], Jacob Zelko[9], Erica Matute-Arcos[1], Mark Abajian[2], Philip R.O. Payne[10], Albert M. Lai[10], Heath A. Davis[11], Asher A. Hoberg[11], Chris E. Ortman[11], Amit Gode[12], Bradley W. Taylor[12], Kristen Osinski[12], Damian N. Di Florio[13-15], Noel R. Rose[16], Frederick W. Miller[17], George C. Tsokos[18], DeLisa Fairweather[13,14,19$#]

[1]Autoimmune Registry Inc., Guilford, Connecticut, USA
[2]Division of Bioinformatics, Department of Population and Public Health Sciences, Keck School of Medicine, University of Southern California, Los Angeles, California, USA
[3]Department of Dermatology, Keck School of Medicine, University of Southern California, Los Angeles, California, USA
[4]University of Florida Health, Gainesville, Florida, USA
[5]Department of Neurology, Massachusetts General Hospital, Boston, Massachusetts, USA
[6]Research Information Systems and Computing, Mass General Brigham, Somerville, Massachusetts, USA
[7]Department of Neurology, University of Kentucky Medical Center, Lexington, Kentucky, USA
[8]Department of Medicine, Weill Cornell Medical Center, New York, New York, USA
[9]Georgia Tech Research Institute, Atlanta, Georgia, USA
[10]Institute for Informatics, Data Science, and Biostatistics, Washington University School of Medicine, St. Louis, Missouri, USA
[11]Institute for Clinical and Translational Science, University of Iowa, Iowa City, Iowa, USA
[12]Clinical & Translational Science Institute, Medical College of Wisconsin, Milwaukee, Wisconsin, USA
[13]Department of Cardiovascular Medicine, Mayo Clinic, Jacksonville, Florida, USA
[14]Center for Clinical and Translational Science, Mayo Clinic, Rochester, Minnesota, USA
[15]Mayo Clinic Graduate School of Biomedical Sciences, Mayo Clinic, Rochester, Minnesota, USA
[16]Harvard Medical School, Boston, Massachusetts, USA
[17]National Institute of Environmental Health Sciences, National Institutes of Health, Research Triangle Park, North Carolina, USA
[18]Department of Medicine, Beth Israel Deaconess Medical Center, Boston, Massachusetts, USA
[19]Department of Immunology, Mayo Clinic, Jacksonville, Florida, USA

[$]Co-Senior authors
[#]Corresponding author

Corresponding author: Dr. DeLisa Fairweather, PhD; Department of Cardiovascular Medicine, Mayo Clinic, 4500 San Pablo Road, Jacksonville, Florida, USA; Tele: +1-904-953-6351; Email: Fairweather.DeLisa@mayo.edu

Supplemental Tables

Supplemental Table 1. List of autoimmune diseases with published prevalence[a]

Autoimmune Disease	Computed Estimated US Prevalence			Female Ratio	Rate/ 100,000	Published Prevalence (Reference)
	Female	Male	Total			
Acquired hemophilia	< 1,000	3,447	3,984	< 22%	1.2	499 (1)
Acute disseminated encephalomyelitis	6,129	5,210	11,339	54%	3.4	1,116 (2)
Acute febrile mucocutaneous lymph node syndrome	11,416	19,691	31,107	37%	9.3	116,321 (3)
Acute hemorrhagic leukoencephalitis	ND[b]	ND	ND	ND	ND	22 (4)
Acute motor axonal neuropathy	ND	ND	ND	ND	ND	823 (5)
Addison's disease	< 1,000	No data	< 2,000	ND	ND	43,778(6)
Adult-onset Still's disease	9,271	5,440	14,711	63%	4.4	1,833 (7)
Alopecia areata	162,435	108,264	270,699	60%	81.2	699,930 (8)
Anti-glomerular basement membrane nephritis	< 1,000	< 1,000	< 2,000	ND	0.3	333 (9)
Anti-neutrophil cytoplasmic antibody-associated vasculitis	17,622	11,646	29,269	60%	8.8	222 (10)
Anti-n-methyl-D-aspartate receptor encephalitis	ND	ND	ND	ND	ND	38,329 (11)
Antiphospholipid syndrome	153,470	56,316	209,786	73%	62.9	149,985 (12)
Antisynthetase syndrome	ND	ND	ND	ND	ND	11,665 (13)
Aplastic anemia	67,196	60,836	128,032	52%	38.4	783 (14)
Atrophic gastritis	86,810	38,616	125,427	69%	37.6	79,992 (15)
Autoimmune encephalitis	2,988	1,762	4,750	63%	1.4	45,662 (16)
Autoimmune enteropathy	ND	ND	ND	ND	ND	100 (17)
Autoimmune hepatitis	150,865	42,754	193,619	78%	58.1	56,327 (18)
Autoimmune inner ear disease	ND	ND	ND	ND	ND	49,995 (19)
Autoimmune lymphoproliferative syndrome	2,605	< 1,000	3,294	< 72%	1	200 (20)
Autoimmune myocarditis	ND	ND	ND	ND	ND	ND
Autoimmune oophoritis	ND	ND	ND	ND	ND	666 (21)
Autoimmune pancreatitis	ND	ND	ND	ND	ND	15,331 (22)
Autoimmune polyendocrine syndrome type 1	2,758	ND	2,758	100%	0.8	ND

Autoimmune polyendocrine syndrome type 2	ND	ND	ND	ND	ND	ND
Autoimmune polyendocrine syndrome type 3	ND	ND	ND	ND	ND	ND
Autoimmune thrombocytopenic purpura	306,251	221,126	527,378	58%	158.2	23,331 (23)
Autoimmune thyroiditis	1,536,929	233,615	1,770,544	87%	531.2	1,833,150 (24)
Autoimmune urticaria	ND	ND	ND	ND	ND	2,666,400 (25)
Autoimmune uveitis	173,851	109,950	283,802	61%	85.2	343,965 (26)
Balo concentric sclerosis	< 1,000	ND	< 2,000	ND	0.1	ND
Behcet's disease	37,237	15,017	52,255	71%	15.7	17,331 (27)
Bickerstaff's encephalitis	ND	ND	ND	ND	ND	ND
Bullous pemphigoid	45,972	37,927	83,899	55%	25.2	86,324 (28)
Celiac disease	514,965	190,171	705,137	73%	211.6	2,916,375 (29)
Chronic inflammatory demyelinating polyneuropathy	39,842	52,025	91,867	43%	27.6	11,232 (30)
Cicatricial pemphigoid	20,534	9,960	30,494	67%	9.1	338 (31)
Cogan syndrome	ND	ND	ND	ND	ND	ND
Cold autoimmune hemolytic anemia	5,823	3,907	9,730	60%	2.9	5,399 (32)
Crohn's disease	620,625	490,064	1,110,689	56%	333.3	2,282,271 (33)
Cryptogenic organizing pneumonia	14,787	12,718	27,506	54%	8.3	3,666 (34)
Cutaneous lupus erythematosus	72,482	10,420	82,903	87%	24.9	253,308 (35)
Dermatitis herpetiformis	6,589	7,662	14,251	46%	4.3	31,163 (36)
Dermatomyositis	108,264	30,418	138,682	78%	41.6	71,392 (37)
Encephalopathy Associated with Autoimmune Thyroid Disease	ND	ND	ND	ND	ND	6,999 (38)
Enthesitis-related arthritis	45,282	35,168	80,451	56%	24.1	ND
Epidermolysis bullosa acquisita	< 1,000	< 1,000	< 2,000	ND	0.1	96 (39)
Episcleritis	40,149	15,553	55,703	72%	16.7	175,315 (40)
Essential mixed cryoglobulinemia	8,504	6,129	14,634	58%	4.4	ND
Evans syndrome	5,746	6,895	12,642	45%	3.8	71,992 (5)
Felty syndrome	2,758	< 1,000	3,218	< 73%	1	33,330 (41)
Gestational pemphigoid	3,984	ND	3,984	100%	1.2	100 (42)
Giant cell arteritis	126,347	54,553	180,900	70%	54.3	235,143 (43)
Granulomatosis with polyangiitis	88,726	59,150	147,877	60%	44.4	53,328 (44)
Graves' disease	1,125,937	314,067	1,440,004	78%	432.1	2,096,457 (45)

Graves' ophthalmopathy	ND	< 1,000	< 2,000	ND	ND	298,959 (46)
Guillain-Barré syndrome	39,842	39,919	79,761	50%	23.9	5,466 (47)
IgA nephropathy	< 1,000	ND	< 2,000	ND	ND	12,665 (48)
Immunoglobulin A vasculitis	21,836	20,381	42,217	52%	12.7	15,034 (49)
Immunoglobulin G4 related disease	ND	ND	ND	ND	ND	2,666 (50)
Inclusion body myositis	11,646	18,695	30,341	38%	9.1	3,333 (51)
Intermediate uveitis	ND	ND	ND	ND	ND	5,666 (14)
Juvenile arthritis	184,272	67,732	252,004	73%	75.6	33,041 (52)
Juvenile dermatomyositis	7,662	2,068	9,730	79%	2.9	7,332 (37)
Lambert-Eaton myasthenic syndrome	2,911	1,302	4,214	69%	1.3	983 (53)
Lichen sclerosus	311,002	14,481	325,483	96%	97.7	166,650 (54)
Linear IgA disease	< 1,000	ND	< 2,000	ND	ND	466 (55)
Lupus nephritis	114,854	21,530	136,384	84%	40.9	70,801 (56)
Lupus vasculitis	ND	ND	ND	ND	ND	26,855 (57)
Microscopic polyangiitis	9,041	2,911	11,952	76%	3.6	31,163 (44)
Mixed connective tissue disease	< 1,000	ND	< 2,000	ND	ND	9,853 (58)
Multiple sclerosis	1,017,672	348,393	1,366,065	74%	409.9	801,496 (59)
Myasthenia gravis	58,154	55,396	113,551	51%	34.1	58,327 (60)
Myelin oligodendrocyte glycoprotein disease	ND	ND	ND	ND	ND	8,365 (61)
Narcolepsy with cataplexy	48,117	25,054	73,172	66%	22	46,662 (62)
Necrotizing vasculitis	14,940	5,286	20,227	74%	6.1	14,998 (63)
Neuromyelitis optica	32,716	10,037	42,754	77%	12.8	8,199 (64)
Neuromyotonia	ND	ND	ND	ND	ND	ND
Opsoclonus myoclonus syndrome	ND	ND	ND	ND	ND	24 (65)
Paraneoplastic cerebellar degeneration	ND	ND	ND	ND	ND	4,078 (66)
Pemphigus vulgaris	39,459	27,889	67,349	59%	20.2	17,164 (67)
Pernicious anemia	40,608	16,626	57,235	71%	17.2	1,645,668 (68)
Polymyalgia rheumatica	413,826	251,314	665,141	62%	199.6	793,820 (43)
Polymyositis	44,976	20,840	65,816	68%	19.7	44,995 (69)
Post-myocardial infarction syndrome	1,225	1,609	2,834	43%	0.9	89,000 (70)
Primary biliary cholangitis	110,793	15,094	125,887	88%	37.8	97,656 (71)
Primary sclerosing cholangitis	23,905	33,559	57,465	42%	17.2	20,281 (72)
Psoriasis	1,290,977	1,047,707	2,338,685	55%	701.7	10,498,950 (73)

Pure red cell aplasia	4,290	4,597	8,887	48%	2.7	ND
Rasmussen disease	ND	ND	ND	ND	ND	ND
Reactive arthritis	9,500	11,646	21,147	45%	6.3	307,135 (74)
Relapsing polychondritis	12,795	4,827	17,622	73%	5.3	1,166 (75)
Rheumatic fever	7,968	3,447	11,416	70%	3.4	16,665 (76)
Rheumatoid arthritis	1,709,171	445,164	2,154,336	79%	646.4	1,866,480 (77)
Rheumatoid vasculitis	9,730	1,149	10,880	89%	3.3	984,901 (78)
Sjögren's disease	628,134	66,966	695,100	90%	208.6	966,570 (79)
Stiff person syndrome	12,948	6,895	19,844	65%	6	333 (80)
Sympathetic ophthalmia	< 1,000	< 1,000	< 2,000	ND	0.4	99 (81)
Systemic lupus erythematosus	988,326	114,241	1,102,567	90%	330.8	243,808 (82)
Systemic scleroderma	194,998	35,934	230,933	84%	69.3	74,992 (83)
Transverse myelitis	16,166	9,117	25,284	64%	7.6	8,199 (84)
Type 1 diabetes mellitus	715,788	634,263	1,350,051	53%	405.1	999,900 (85)
Ulcerative colitis	502,246	421,795	924,042	54%	277.3	1,867,313 (33)
Vitiligo	159,906	130,714	290,621	55%	87.2	499,950 (86)
Vogt–Koyanagi–Harada syndrome	7,355	2,681	10,037	73%	3	1,249 (87)
Warm autoimmune hemolytic anemia	70,337	50,799	121,136	58%	36.3	39,996 (24)

[a] Data from Mass General Brigham, University Florida/Shands and University of Southern California

[b] ND, no data- no published papers available or too few patients (less than 20 patients with the condition from the 3 sites) to be able to determine prevalence.

Supplemental Table 2. Prevalence of diseases considered but not included in the analysis due to inadequate evidence of autoimmune disease

	Computed Estimated US Prevalence					
**Disease**	**Female**	**Male**	**Total**	**Female Ratio**	**Rate/ 100,000**	**Published Prevalence (Reference)**
Adiposis dolorosa	ND [b]	ND	ND	ND	ND	ND (88)
Age related macular degeneration	326,939	202,584	529,523	62%	158.9	ND
Ankylosing spondylitis	120,140	182,433	302,574	40%	90.8	866,580 (89)
Anti-sperm antibodies	ND	ND	ND	ND	ND	ND
Autoimmune angioedema	ND	ND	ND	ND	ND	1,999 (90)
Autoimmune neutropenia	ND	ND	ND	ND	ND	1,666 (91)
Autoimmune orchitis	ND	ND	ND	ND	ND	ND
Autoimmune progesterone dermatitis	ND	ND	ND	ND	ND	ND
Autoimmune retinopathy	ND	ND	ND	ND	ND	ND
Brachial neuropathy	442,942	286,253	729,196	61%	218.8	5,466 (92)
Chronic lyme disease	202,201	153,470	355,672	57%	106.7	ND
Chronic recurring multifocal osteomyelitis	12,565	17,316	29,881	42%	9.0	333 (93)
Complex regional pain syndrome	153,700	51,565	205,266	75%	61.6	68,559 (94)
Cutaneous mastocytosis	17,699	11,263	28,962	61%	8.7	33,330 (95)
Endometriosis	1,001,352	< 1,000	1,001,505	N/A	300.5	806,250 (96)
Eosinophilic esophagitis	154,236	259,743	413,980	37%	124.2	111,655 (97)
Eosinophilic fasciitis	4,980	2,528	7,508	66%	2.3	5,166 (98)
Epilepsy	1,839,886	1,661,054	3,500,940	53%	1,050.4	ND
Erythema nodosum	36,241	6,053	42,294	86%	12.7	9,999 (99)
Fibromyalgia	1,704,727	106,732	1,811,460	94%	543.5	5,816,085 (100)
Hidradenitis suppurativa	309,776	88,419	398,196	78%	119.5	2,283,105 (101)
Idiopathic pulmonary fibrosis	68,651	123,971	192,623	36%	57.8	1,161,050 (102)
Interstitial cystitis	194,692	17,929	212,621	92%	63.8	55,161 (103)
Lichen planus	188,409	61,066	249,476	76%	74.9	ND
Ligneous conjunctivitis	ND	< 1,000	< 2,000	ND	ND	ND
Microscopic colitis	40,762	11,569	52,331	78%	15.7	343,299 (104)
Mooren's ulcer	< 1,000	< 1,000	< 2,000	ND	0.2	ND
Morphea	107,038	12,029	119,068	90%	35.7	5,166 (105)

Myalgic encephalomyelitis	1,324,843	586,835	1,911,679	69%	573.6	7,599,240 (106)
Myositis	397,966	114,011	511,977	78%	153.6	47,161 (107)
Palindromic rheumatism	12,412	6,206	18,618	67%	5.6	ND
Paroxysmal nocturnal hemoglobinuria	7,815	6,359	14,174	55%	4.3	416 (108)
Parry Romberg syndrome	ND	ND	ND	ND	ND	476 (109)
Pediatric autoimmune neuropsychiatric disorder associated with streptococcus	ND	ND	ND	ND	ND	370,000 (110)
Pityriasis lichenoides et varioliformis acuta	3,984	< 1,000	4,903	< 80%	1.5	101,729
POEMS syndrome	< 1,000	ND	< 2,000	ND	ND	999 (111)
Polyarteritis nodosa	15,477	10,650	26,127	59%	7.8	533 (112)
Post-acute sequelae of COVID-19	ND	ND	ND	ND	ND	ND
Primary idiopathic dilated cardiomyopathy	ND	ND	ND	ND	ND	121,654 (113)
Psoriatic arthritis	1,302	1,072	2,375	55%	0.7	516,615 (114)
Pyoderma gangrenosum	28,809	11,033	39,842	72%	12.0	19,498 (115)
Restless leg syndrome	993,996	517,264	1,511,261	66%	453.4	54,994,500 (116)
Retinocochleocerebral vasculopathy	ND	ND	ND	ND	ND	ND
Retroperitoneal fibrosis	ND	ND	ND	ND	ND	4,666 (117)
Rheumatic chorea	< 1,000	< 1,000	1,072	ND	0.3	ND
Sarcoidosis	350,615	255,758	606,374	58%	181.9	198,680 (118)
Schnitzler syndrome	ND	< 1,000	< 2,000	ND	ND	300 (119)
Scleritis	88,113	38,923	127,036	69%	38.1	17,331 (40)
Secondary Raynaud's phenomenon	15,707	1,302	17,009	92%	5.1	17,981,535 (120)
Subacute bacterial endocarditis	7,508	10,803	18,312	41%	5.5	21,664 (121)
Systemic mast cell disease	164,657	34,938	199,596	82%	59.9	ND
Takayasu arteritis	19,308	3,064	22,373	86%	6.7	1,566 (122)
Tolosa-Hunt syndrome	ND	ND	ND	ND	ND	300 (123)
Undifferentiated connective tissue disease	153	ND	< 2,000	ND	ND	256,641 (124)

[a] Data from Mass General Brigham, University Florida/Shands and University of Southern California
[b] ND, no data- no published papers available or too few patients (less than 20 patients with the condition from the 3 sites) to be able to determine prevalence.

Supplemental Table 3. US Census population in 2022

	US Census Population in 2022		
**Age**	**Female**	**Male**	**Total**
0-17	35,271,925	37,053,677	72,325,602
18-44	59,242,372	61,293,359	120,535,731
45-64	41,647,380	40,956,534	82,603,914
≥65	31,897,671	25,924,644	57,822,315
**Total**	168,059,348	165,228,214	333,287,562

Supplemental Table 4. Number of patients with one or more autoimmune diseases

Number of Autoimmune Diseases	USC[a]	UF	MGB	WUSL	UI	MCW	Total	Percent
**1**	10,894	32,031	110,401	121,782	43,049	61,267	379,424	65%
**2**	8,629	21,893	30,425	43,771	21,368	15,591	141,677	24%
**3**	4,539	9,982	7,293	10,723	9,015	3,794	45,346	8%
**4+**	1,958	2,182	2,784	4,580	692	1,510	13,706	2%
**Total**	26,020	66,088	150,903	180,856	74,124	82,162	580,153	

[a]Abbreviations: MCW, Medical College of Wisconsin; MGB, Mass General Brigham; UF, University Florida/Shands; UI, University of Iowa; USC, University of Southern California; WUSL, University of Washington at St. Louis

Supplementary Table 5. Analysis of diagnosis code frequency (numerator) and timing (denominator)

Autoimmune Disease	0 days	1 day	7 days	30 days	90 days	365 days	30 vs. 0 days[a]
Antiphospholipid syndrome (hematologic)	739	418	396	359	320	208	51%
Crohn's disease (digestive)	1,898	1,210	1,125	1,023	900	659	46%
Multiple sclerosis (neurologic)	4,035	3,097	2,953	2,786	2,594	2,107	31%
Rheumatoid arthritis (connective tissue)	8,101	4,915	4,676	4,314	3,831	2,811	47%
Systemic lupus erythematosus (systemic)	4,691	2,970	2,845	2,638	2,347	1,703	44%
Vitiligo (skin)	883	538	479	413	368	244	53%
**Any of these 6 diseases**	**18,498**	**12,354**	**11,723**	**10,857**	**9,777**	**7,370**	**41%**

[a]This column shows the ratio that a 0-day algorithm overstates the number of cases compared to the 30-day algorithm for each disease.

Supplementary Table 6. Date-window sensitivity analysis (USC data)[a]

Date-window: 0

	Autoimmune Disease Counts/ Denominators (%)		Projected Prevalence[a]		
**Age**	**Female**	**Male**	**Female**	**Male**	**Total**
0-17	73/9,439 (0.77%)	85/10,604 (0.80%)	272,788	297,016	569,805
18-44	8,042/91,017 (8.84%)	4,323/76,816 (5.63%)	5,234,485	3,449,427	8,683,912
45-64	11,317/87,591 (12.92%)	5,922/79,350 (7.46%)	5,380,957	3,056,643	8,437,600
≥65	13,235/111,661 (11.85%)	7,940/113,471 (7.00%)	3,780,780	1,814,047	5,594,827
**Total**			14,669,011	8,617,133	**23,286,144 (6.99%)**

Date-window: 30

	Autoimmune Disease Counts/ Denominators (%)		Projected Prevalence[a]		
**Age**	**Female**	**Male**	**Female**	**Male**	**Total**
0-17	18/3,571 (0.05%)	23/4,098 (0.06%)	177,792	207,964	385,755
18-44	4,542/53,306 (8.52%)	2,350/43,826 (5.36%)	5,047,816	3,286,620	8,334,435
45-64	6,351/58,371 (10.88%)	3,025/52,288 (5.79%)	4,531,403	2,369,445	6,900,847
≥65	7,189/78,373 (9.17%)	3,712/80,614 (4.61%)	2,925,910	1,193,742	4,119,652
**Total**			12,682,920	7,057,769	**19,740,690 (5.92%)**

Date-window: 60

	Autoimmune Disease Counts/ Denominators (%)		Projected Prevalence[a]		
**Age**	**Female**	**Male**	**Female**	**Male**	**Total**
0-17	18/3,254 (0.06%)	19/3,733 (0.05%)	195,112	188,594	383,706
18-44	4,219 /47,914 (8.81%)	2,146 /38,725 (5.54%)	5,216,504	3,396,657	8,613,161
45-64	5,948 /53,665 (11.08%)	2,786 /47,337 (5.89%)	4,616,018	2,410,480	7,026,498
≥65	6,691 /72,130 (9.28%)	3,400 /73,695 (4.61%)	2,958,926	1,196,062	4,154,988
**Total**			12,986,560	7,191,793	**20,178,353 (6.05%)**

Date-window: 90

	Autoimmune Disease Counts/ Denominators (%)		Projected Prevalence[a]		
**Age**	**Female**	**Male**	**Female**	**Male**	**Total**
0-17	17/3,071 (0.06%)	19/3,526 (0.05%)	195,253	199,665	394,919
18-44	3,971 /44,415 (8.94%)	2,010 /35,433 (5.67%)	5,296,667	3,476,975	8,773,642
45-64	5,646 /50,422 (11.19%)	2,631 /44,074 (5.97%)	4,663,463	2,444,903	7,108,365
≥65	6,315 /67,810 (9.28%)	3,213 /68,939 (4.66%)	2,970,562	1,208,255	4,178,817
**Total**			13,125,944	7,329,798	**20,455,742 (6.14%)**

Date-window: 180

	Autoimmune Disease Counts/ Denominators (%)		Projected Prevalence[a]		
**Age**	**Female**	**Male**	**Female**	**Male**	**Total**
0-17	14/2,747 (0.05%)	15/3,135 (0.05%)	179,762	177,290	357,053
18-44	3,466/37,727 (9.19%)	1,739/29,385 (5.92%)	5,442,629	3,627,332	9,069,961
45-64	5,014/44,046 (11.38%)	2,285/37,670 (6.07%)	4,740,952	2,484,356	7,225,308
≥65	5,571/59,376 (9.38%)	2,763/59,724 (4.63%)	2,992,824	1,199,347	4,192,171
**Total**			13,356,167	7,488,325	**20,844,492 (6.25%)**

Date-window: 365

	Autoimmune Disease Counts/ Denominators (%)		Projected Prevalence[a]		
**Age**	**Female**	**Male**	**Female**	**Male**	**Total**
0-17	11/2,234 (0.05%)	10/2,507 (0.04%)	173,676	147,801	321,476
18-44	2,773 /29,463 (9.41%)	1,355 /22,357 (6.06%)	5,575,776	3,714,832	9,290,608
45-64	4,209 /36,131 (11.65%)	1,897 /30,100 (6.30%)	4,851,618	2,581,214	7,432,832
≥65	4,646 /49,058 (9.47%)	2,209 /48,502 (4.55%)	3,020,844	1,180,725	4,201,570
**Total**			13,621,915	7,624,572	**21,246,487 (6.37%)**

Date-window: 720

	Autoimmune Disease Counts/ Denominators (%)		Projected Prevalence[a]		
**Age**	**Female**	**Male**	**Female**	**Male**	**Total**
0-17	5/1,548 (0.03%)	4/1,723 (0.02%)	113,927	86,021	199,949
18-44	1,926 /20,109 (9.58%)	966 /14,994 (6.44%)	5,674,116	3,948,872	9,622,988
45-64	3,221 /27,195 (11.84%)	1,425 /21,900 (6.51%)	4,932,753	2,664,980	7,597,733
≥65	3,532 /37,367 (9.45%)	1,598 /35,801 (4.46%)	3,015,029	1,157,163	4,172,191
**Total**			13,735,825	7,857,036	**21,592,861 (6.48%)**

[a]Based-on US Census Data for 2022; sex and age adjusted

Supplementary Table 7. Percent change using different date windows[a]

Date-Window	Prevalence[b]	Prevalence % US Population[b]	% Change
**0**	23,286,144	6.99%	
**30**	19,740,690	5.92%	-17.96%
**60**	20,178,353	6.05%	2.17%
**90**	20,455,742	6.14%	1.36%
**180**	20,844,492	6.25%	1.87%
**365**	21,246,487	6.37%	1.89%
**720**	21,592,861	6.48%	1.60%

[a]Sex and age adjusted; [b]data from Supplementary Table 6

Supplementary Table 8. Validation of algorithm for rheumatoid arthritis (USC data)[a]

Algorithm[b]	Site	Numerator	Denominator	Projected US Prevalence	% Prevalence
**Harvard**	USC[a]	2,552	375,253	2,266,604	6.80%
**Ours**[c]	USC	2,912	375,253	2,586,344	7.76%
**Ours**	All 6 sites	80,984	10,461,368	2,580,060	7.74%

[a]Abbreviations: USC, University of Southern California; [b]Phenotype 585 (https://phekb.org/phenotype/rheumatoid-arthritis-ra); [c]Age adjusted, single site

References

1. Collins PW, et al. Acquired hemophilia A in the United Kingdom: A 2-year national surveillance study by the United Kingdom Haemophilia Centre Doctors' Organisation. *Blood*. 2007;109(5):1870-1877.

2. Filippi M, Rocca, M.A. *Acute Disseminated Encephalomyelitis*. White Matter Diseases. Springer, Cham; 2020.

3. Wu MH, et al. Prevalence and the long-term coronary risks of patients with Kawasaki disease in a general population <40 years: A national database study. *Circ Cardiovasc Qual Outcomes*. 2012;5(4):566-750.

4. Tenembaum S, et al. Acute disseminated encephalomyelitis: a long-term follow-up study of 84 pediatric patients. *Neurology*. 2002;59(8):1224-1231.

5. Hansen DL, et al. Evans syndrome in adults - Incidence, prevalence, and survival in a nationwide cohort. *Am J Hematol*. 2019;94(10):1081-1090.

6. Olafsson AS, Sigurjonsdottir HA. Increasing prevalence of Addison Disease: Results from a nationwide study. *Endocr Pract*. 2016;22(1):30-35.

7. Mitrovic S, Feist E, Fautrel B, Eds. *Adult-Onset Still's Disease*. Periodic and Non-Periodic Fevers Rare Diseases of the Immune System. Springer, Cham; 2020.

8. Benigno M, et al. A large cross-sectional survey study of the prevalence of alopecia areata in the United States. *Clin Cosmet Investig Dermatol*. 2020;13:259-266.

9. Lingaraj U, et al. A "Mini-Epidemic" of anti-glomerular basement membrane disease: Clinical and epidemiological study. *Saudi J Kidney Dis Transpl*. 2017;28(5):1057-1063.

10. Berti A, et al. The epidemiology of antineutrophil cytoplasmic autoantibody-associated vasculitis in Olmsted County, Minnesota: A twenty-year US population-based study. *Arthritis Rheumatol*. 2017;69(12):2338-2350.

11. Samanta D, Lui F. Anti-NMDAR Encephalitis. *StatPearls*. 2023.

12. Gomez-Puerta JA, Cervera R. Diagnosis and classification of the antiphospholipid syndrome. *J Autoimmun*. 2014;48-49:20-25.

13. Cojocaru M, et al. New insights into antisynthetase ayndrome. *Maedica (Bucur)*. 2016;11(2):130-135.

14. Vaht K, et al. Incidence and outcome of acquired aplastic anemia: Real-world data from patients diagnosed in Sweden from 2000-2011. *Haematologica*. 2017;102(10):1683-1690.

15. Jensen ET, et al. Prevalence of eosinophilic gastritis, gastroenteritis, and colitis: Estimates from a national administrative database. *J Pediatr Gastroenterol Nutr*. 2016;62(1):36-42.

16. Dubey D, et al. Autoimmune encephalitis epidemiology and a comparison to infectious encephalitis. *Ann Neurol*. 2018;83(1):166-177.

17. Ruemmele FM. *Chapter 85 - Autoimmune Enteropathy and IPEX Syndrome*. 4th ed. vol 2. Academic Press; 2015.

18. Boberg KM, et al. Incidence and prevalence of primary biliary cirrhosis, primary sclerosing cholangitis, and autoimmune hepatitis in a Norwegian population. *Scand J Gastroenterol*. 1998;33(1):99-103.

19. Vambutas A, Pathak S. AAO: Autoimmune and autoinflammatory (sisease) in otology: What is new in immune-mediated hearing loss. *Laryngoscope Investig Otolaryngol*. 2016;1(5):110-115.

20. Shah S, et al. Autoimmune lymphoproliferative syndrome: An update and review of the literature. *Curr Allergy Asthma Rep*. 2014;14(9):462.

21. Tsigkou A, et al. High serum inhibin concentration discriminates autoimmune oophoritis from other forms of primary ovarian insufficiency. *J Clin Endocrinol Metab*. 2008;93(4):1263-1269.

22. Cai O, Tan S. From pathogenesis, clinical manifestation, and diagnosis to treatment: An overview on autoimmune pancreatitis. *Gastroenterol Res Pract*. 2017;2017:3246459.

23. Segal JB, Powe NR. Prevalence of immune thrombocytopenia: Analyses of administrative data. *J Thromb Haemost*. 2006;4(11):2377-2383.

24. Eaton WW, et al. The prevalence of 30 ICD-10 autoimmune diseases in Denmark. *Immunol Res*. 2010;47(1-3):228-231.

25. Zuberbier T, et al. Epidemiology of urticaria: A representative cross-sectional population survey. *Clin Exp Dermatol*. 2010;35(8):869-873.

26. Acharya NR, et al. Incidence and prevalence of uveitis: Results from the Pacific Ocular Inflammation Study. *JAMA Ophthalmol*. 2013;131(11):1405-1412.

27. Calamia KT, et al. Epidemiology and clinical characteristics of Behcet's disease in the US: A population-based study. *Arthritis Rheum*. 2009;61(5):600-604.

28. Kridin K, Ludwig RJ. The growing incidence of bullous pemphigoid: Overview and potential explanations. *Front Med (Lausanne)*. 2018;5:220.

29. Gujral N, et al. Celiac disease: Prevalence, diagnosis, pathogenesis and treatment. *World J Gastroenterol*. 2012;18(42):6036-6059.

30. Rajabally YA, et al. Epidemiologic variability of chronic inflammatory demyelinating polyneuropathy with different diagnostic criteria: Study of a UK population. *Muscle Nerve*. 2009;39(4):432-438.

31. Tolaymat L, Hall MR. Cicatricial Pemphigoid. *StatPearls*. 2023.

32. Mullins M, et al. Cold agglutinin disease burden: A longitudinal analysis of anemia, medications, transfusions, and health care utilization. *Blood Adv*. 2017;1(13):839-848.

33. Dahlhamer JM, et al. Prevalence of inflammatory bowel disease among adults aged ≥18 years - United States, 2015. *MMWR Morb Mortal Wkly Rep*. 2016;65(42):1166-1169.

34. Gudmundsson G, et al. Epidemiology of organising pneumonia in Iceland. *Thorax*. 2006;61(9):805-808.

35. Gauzere L, et al. Epidemiology of systemic lupus erythematosus in Reunion Island, Indian Ocean: A case-series in adult patients from a University Hospital. *Rev Med Interne*. 2019;40(4):214-219. [Caracteristiques du lupus erythemateux systemique a La Reunion : etude retrospective en population adulte au CHU de Saint-Denis.]

36. Shields BE, et al. Prevalence of dermatitis herpetiformis within the iCureCeliac Patient-Powered Research Network- Patient characteristics and dietary counseling. *JAMA Dermatol*. 2020;156(12):1374-1376.

37. Bendewald MJ, et al. Incidence of dermatomyositis and clinically amyopathic dermatomyositis: A population-based study in Olmsted County, Minnesota. *Arch Dermatol*. 2010;146(1):26-30.

38. Mocellin R, et al. Hashimoto's encephalopathy: Epidemiology, pathogenesis and management. *CNS Drugs*. 2007;21(10):799-811.

39. Iranzo P, et al. Epidermolysis bullosa acquisita: A retrospective analysis of 12 patients evaluated in four tertiary hospitals in Spain. *Br J Dermatol*. 2014;171(5):1022-1030.

40. Honik G, et al. Incidence and prevalence of episcleritis and scleritis in Northern California. *Cornea*. 2013;32(12):1562-1566.

41. Calguneri M, et al. Extra-articular manifestations of rheumatoid arthritis: Results of a university hospital of 526 patients in Turkey. *Clin Exp Rheumatol*. 2006;24(3):305-308.

42. Fong M, et al. Pemphigoid Gestationis. *StatPearls*. 2023.

43. Crowson CS, Matteson EL. Contemporary prevalence estimates for giant cell arteritis and polymyalgia rheumatica, 2015. *Semin Arthritis Rheum*. 2017;47(2):253-256.

44. Mohammad AJ, et al. Prevalence of Wegener's granulomatosis, microscopic polyangiitis, polyarteritis nodosa and Churg-Strauss syndrome within a defined population in southern Sweden. *Rheumatology (Oxford)*. 2007;46(8):1329-1337.

45. Eaton WW, et al. Epidemiology of autoimmune diseases in Denmark. *J Autoimmun*. 2007;29(1):1-9.

46. Bartalena L, Tanda ML. Clinical practice. Graves' ophthalmopathy. *N Engl J Med*. 2009;360(10):994-1001.

47. Sejvar JJ, et al. Population incidence of Guillain-Barre syndrome: A systematic review and meta-analysis. *Neuroepidemiology*. 2011;36(2):123-133.

48. Schena FP, Nistor I. Epidemiology of IgA nephropathy: A global perspective. *Semin Nephrol*. 2018;38(5):435-442.

49. Piram M, Mahr A. Epidemiology of immunoglobulin A vasculitis (Henoch-Schonlein): Current state of knowledge. *Curr Opin Rheumatol*. 2013;25(2):171-178.

50. Oprita R, et al. Overview of IgG4 - related disease. *J Med Life*. 2017;10(4):203-207.

51. Greenberg SA. Inclusion body myositis: Clinical features and pathogenesis. *Nat Rev Rheumatol*. 2019;15(5):257-272.

52. Harrold LR, et al. Incidence and prevalence of juvenile idiopathic arthritis among children in a managed care population, 1996-2009. *J Rheumatol*. 2013;40(7):1218-25.

53. Abenroth DC, et al. Lambert-Eaton myasthenic syndrome: Epidemiology and therapeutic response in the national veterans affairs population. *Muscle Nerve*. 2017;56(3):421-426.

54. Melnick LE, et al. Lichen sclerosus among women in the United States. *Int J Womens Dermatol*. 2020;6(4):260-262.

55. Fortuna G, Marinkovich MP. Linear immunoglobulin A bullous dermatosis. *Clin Dermatol*. 2012;30(1):38-50.

56. Wang H, et al. A systematic review and meta-analysis of prevalence of biopsy-proven lupus nephritis. *Arch Rheumatol*. 2018;33(1):17-25.

57. Ramos-Casals M, et al. Vasculitis in systemic lupus erythematosus: Prevalence and clinical characteristics in 670 patients. *Medicine (Baltimore)*. 2006;85(2):95-104.

58. Gunnarsson R, et al. The prevalence and incidence of mixed connective tissue disease: A national multicentre survey of Norwegian patients. *Ann Rheum Dis*. 2011;70(6):1047-1051.

59. Wallin MT, et al. The prevalence of MS in the United States: A population-based estimate using health claims data. *Neurology*. 2019;92(10):e1029-e1040.

60. Keesey JC. Clinical evaluation and management of myasthenia gravis. *Muscle Nerve*. 2004;29(4):484-505.

61. Hor JY, Fujihara K. Epidemiology of myelin oligodendrocyte glycoprotein antibody-associated disease: A review of prevalence and incidence worldwide. *Front Neurol*. 2023;14:1260358.

62. Scheer D, et al. Prevalence and incidence of narcolepsy in a US health care claims database, 2008-2010. *Sleep*. 2019;42(7):zsz091.

63. Arora A, et al. Incidence of leukocytoclastic vasculitis, 1996 to 2010: A population-based study in Olmsted County, Minnesota. *Mayo Clin Proc*. 2014;89(11):1515-2154.

64. Marrie RA, Gryba C. The incidence and prevalence of neuromyelitis optica: a systematic review. *Int J MS Care*. 2013;15(3):113-118.

65. Pranzatelli MR, et al. Demographic, clinical, and immunologic features of 389 children with Opsoclonus-Myoclonus Syndrome: A cross-sectional study. *Front Neurol*. 2017;8:468.

66. Vogrig A, et al. Epidemiology of paraneoplastic neurological syndromes: A population-based study. *J Neurol*. 2020;267(1):26-35.

67. Wertenteil S, et al. Prevalence estimates for pemphigus in the United States: A sex- and age-adjusted population analysis. *JAMA Dermatol*. 2019;155(5):627-629.

68. Toh BH, et al. Pernicious anemia. *N Engl J Med*. 1997;337(20):1441-1448.

69. Cheeti A, et al. Autoimmune Myopathies. *StatPearls*. 2023.

70. Miller RH, et al. The epidemiology of the postpericardiotomy syndrome: A common complication of cardiac surgery. *Am Heart J*. 1988;116(5 Pt 1):1323-1329.

71. Lu M, et al. Factors associated with prevalence and treatment of primary biliary cholangitis in United States Health Systems. *Clin Gastroenterol Hepatol*. 2018;16(8):1333-1341.e6.

72. Liang H, et al. Incidence, prevalence, and natural history of primary sclerosing cholangitis in the United Kingdom. *Medicine (Baltimore)*. 2017;96(24):e7116.

73. Rachakonda TD, et al. Psoriasis prevalence among adults in the United States. *J Am Acad Dermatol*. 2014;70(3):512-516.

74. Hanova P, et al. Incidence and prevalence of psoriatic arthritis, ankylosing spondylitis, and reactive arthritis in the first descriptive population-based study in the Czech Republic. *Scand J Rheumatol*. 2010;39(4):310-317.

75. Kent PD, et al. Relapsing polychondritis. *Curr Opin Rheumatol*. 2004;16(1):56-61.

76. Lahiri S, Sanyahumbi A. Acute rheumatic fever. *Pediatr Rev*. 2021;42(5):221-232.

77. Tuncer T, et al. Prevalence of rheumatoid arthritis and spondyloarthritis in Turkey: A nationwide study. *Arch Rheumatol*. 2018;33(2):128-136.

78. Bartels C, et al. Decline in rheumatoid vasculitis prevalence among US veterans: A retrospective cross-sectional study. *Arthritis Rheum*. 2009;60(9):2553-2557.

79. Narvaez J, et al. Prevalence of Sjogren's syndrome in the general adult population in Spain: Estimating the proportion of undiagnosed cases. *Sci Rep*. 2020;10(1):10627.

80. Nguyen A, et al. Chronic intestinal pseudo-obstruction with dilated biliary tract as a spectrum of stiff person syndrome in a nondiabetic patient. *J Musculoskelet Neuronal Interact*. 2019;19(4):526-530.

81. Arevalo JF, et al. Update on sympathetic ophthalmia. *Middle East Afr J Ophthalmol*. 2012;19(1):13-21.

82. Izmirly PM, et al. Prevalence of systemic lupus erythematosus in the United States: Estimates from a meta-analysis of the Centers for Disease Control and Prevention National Lupus Registries. *Arthritis Rheumatol*. 2021;73(6):991-996.

83. Barnes J, Mayes MD. Epidemiology of systemic sclerosis: Incidence, prevalence, survival, risk factors, malignancy, and environmental triggers. *Curr Opin Rheumatol*. 2012;24(2):165-170.

84. Holroyd KB, et al. Prevalence and characteristics of transverse myelitis and neuromyelitis optica spectrum disorders in the United Arab Emirates: A multicenter, retrospective study. *Clin Exp Neuroimmunol*. 2018;9(3):155-161.

85. Menke A, et al. The prevalence of type 1 diabetes in the United States. *Epidemiology*. 2013;24(5):773-774.

86. Zhang Y, et al. The prevalence of vitiligo: A meta-analysis. *PLoS One*. 2016;11(9):e0163806.

87. Lavezzo MM, et al. Vogt-Koyanagi-Harada disease: Review of a rare autoimmune disease targeting antigens of melanocytes. *Orphanet J Rare Dis*. 2016;11:29.

88. Cook JC, Gross GP. Adiposis Dolorosa. *StatPearls*. 2023.

89. Bakland G, et al. Incidence and prevalence of ankylosing spondylitis in Northern Norway. *Arthritis Rheum*. 2005;53(6):850-855.

90. Swanson TJ, Patel BC. Acquired Angioedema. *StatPearls*. 2023.

91. Chaudhari PM, Mukkamalla SKR. Autoimmune and Chronic Neutropenia. *StatPearls*. 2023.

92. Ohta R, Shimabukuro A. Parsonage-Turner syndrome in a patient with bilateral shoulder pain: A case report. *J Rural Med*. 2017;12(2):135-138.

93. Buch K, et al. Chronic non-bacterial osteomyelitis: A review. *Calcif Tissue Int*. 2019;104(5):544-553.

94. Sandroni P, et al. Complex regional pain syndrome type I: Incidence and prevalence in Olmsted county, a population-based study. *Pain*. 2003;103(1-2):199-207.

95. Di Raimondo C, et al. Cutaneous mastocytosis: A dermatological perspective. *Australas J Dermatol*. 2021;62(1):e1-e7.

96. Eisenberg VH, et al. Epidemiology of endometriosis: A large population-based database study from a healthcare provider with 2 million members. *BJOG*. 2018;125(1):55-62.

97. Moawad FJ. Eosinophilic esophagitis: Incidence and prevalence. *Gastrointest Endosc Clin N Am*. 2018;28(1):15-25.

98. Spielmann L, et al. Population-based prevalence of eosinophilic fasciitis (Shulman syndrome): A capture-recapture study. *Br J Dermatol*. 2018;179(2):516-517.

99. Requena L, Yus ES. Panniculitis. Part I. Mostly septal panniculitis. *J Am Acad Dermatol*. 2001;45(2):163-183; quiz 184-186.

100. Walitt B, et al. The prevalence and characteristics of fibromyalgia in the 2012 National Health Interview Survey. *PLoS One*. 2015;10(9):e0138024.

101. Calao M, et al. Hidradenitis suppurativa (HS) prevalence, demographics and management pathways in Australia: A population-based cross-sectional study. *PLoS One*. 2018;13(7):e0200683.

102. Raghu G, et al. Idiopathic pulmonary fibrosis in US Medicare beneficiaries aged 65 years and older: Incidence, prevalence, and survival, 2001-11. *Lancet Respir Med*. 2014;2(7):566-572.

103. Patnaik SS, et al. Etiology, pathophysiology and biomarkers of interstitial cystitis/painful bladder syndrome. *Arch Gynecol Obstet*. 2017;295(6):1341-1359.

104. Pardi DS, et al. The epidemiology of microscopic colitis: A population based study in Olmsted County, Minnesota. *Gut*. 2007;56(4):504-508.

105. Florez-Pollack S, et al. Morphea: Current concepts. *Clin Dermatol*. 2018;36(4):475-486.

106. Johnston S, et al. The prevalence of chronic fatigue syndrome/ myalgic encephalomyelitis: A meta-analysis. *Clin Epidemiol*. 2013;5:105-110.

107. Meyer A, et al. Incidence and prevalence of inflammatory myopathies: A systematic review. *Rheumatology (Oxford)*. 2015;54(1):50-63.

108. Jalbert JA, Chaudhari, U., Zhang, H., Weyne, J., Shammo, J.M. Epidemiology of PNH and real-world treatment patterns following an incident PNH diagnosis in the US. *Blood*. 2019;134(1):3407.

109. Gupta R, Patil H. Parry-Romberg syndrome with multiple intracranial cysts: A rare case report. *J Pediatr Neurosci*. 2016;11(2):145-149.

110. Wald ER, et al. Estimate of the incidence of PANDAS and PANS in 3 primary care populations. *Front Pediatr*. 2023;11:1170379.

111. Wang Y, et al. Characteristics of 1946 cases of POEMS Syndrome in Chinese subjects: A literature-based study. *Front Immunol*. 2019;10:1428.

112. Hernandez-Rodriguez J, et al. Diagnosis and classification of polyarteritis nodosa. *J Autoimmun*. 2014;48-49:84-89.

113. Codd MB, et al. Epidemiology of idiopathic dilated and hypertrophic cardiomyopathy. A population-based study in Olmsted County, Minnesota, 1975-1984. *Circulation*. 1989;80(3):564-572.

114. Ogdie A, Weiss P. The epidemiology of psoriatic arthritis. *Rheum Dis Clin North Am*. 2015;41(4):545-568.

115. Xu A, et al. Prevalence estimates for pyoderma gangrenosum in the United States: An age- and sex-adjusted population analysis. *J Am Acad Dermatol*. 2020;83(2):425-429.

116. Innes KE, et al. Prevalence of restless legs syndrome in North American and Western European populations: A systematic review. *Sleep Med*. 2011;12(7):623-634.

117. Uibu T, et al. Asbestos exposure as a risk factor for retroperitoneal fibrosis. *Lancet*. 2004;363(9419):1422-1426.

118. Baughman RP, et al. Sarcoidosis in America. Analysis based on health care use. *Ann Am Thorac Soc*. 2016;13(8):1244-1252.

119. de Koning HD. Schnitzler's syndrome: Lessons from 281 cases. *Clin Transl Allergy*. 2014;4:41.

120. Garner R, et al. Prevalence, risk factors and associations of primary Raynaud's phenomenon: Systematic review and meta-analysis of observational studies. *BMJ Open*. 2015;5(3):e006389.

121. Cahill TJ, et al. Challenges in infective endocarditis. *J Am Coll Cardiol*. 2017;69(3):325-344.

122. Watts R, et al. The epidemiology of Takayasu arteritis in the UK. *Rheumatology (Oxford)*. 2009;48(8):1008-1011.

123. Iaconetta G, et al. Tolosa-Hunt syndrome extending in the cerebello-pontine angle. *Cephalalgia*. 2005;25(9):746-750.

124. Spinillo A, et al. Undifferentiated connective tissue diseases and adverse pregnancy outcomes. An undervalued association? *Am J Reprod Immunol*. 2017;78(6) doi: 10.1111/aji.12762.
